# A Case of Spurting Bleeding After Endoscopic Papillary Balloon Dilation

**DOI:** 10.1016/j.gastha.2023.07.005

**Published:** 2023-07-18

**Authors:** Kento Shionoya, Kazuya Koizumi, Sakue Masuda, Jun Kubota, Karen Kimura, Makomo Makazu

**Affiliations:** Gastroenterology Medicine Center, Shonan Kamakura General Hospital, Kamakura-shi, Kanagawa, Japan

**Keywords:** Common bile duct stone, EPBD, ERCP, EST, Prasugrel, Warfarin

## Abstract

A 58-year-old male with acute cholangitis due to a common bile duct stone underwent endoscopic retrograde cholangiopancreatography for stone removal with endoscopic papillary balloon dilation (EPBD) due to his high bleeding risk owing to maintenance dialysis and antiplatelet and anticoagulant medications. He had a history of stone removal using an EPBD. The stone was removed; however, the patient subsequently developed spurting bleeding and underwent endoscopic hemostasis. Despite the subsequent mild pancreatitis, he recovered with conservative management. While EPBD is considered a low-risk procedure for bleeding, caution should still be exercised due to the possibility of massive postprocedural bleeding.

## Introduction

Endoscopic sphincterotomy (EST) is a standard transpapillary procedure for bile duct stones.^1^^,^^2^ Endoscopic papillary balloon dilation (EPBD) is another technique used for stone removal, especially in patients with bleeding tendencies, because it is associated with lower postoperative bleeding rates.^3^, ^4^, ^5^

Herein, we report a case of spurting bleeding after EPBD in a patient with a high bleeding risk.

## Case report

**Figure 1:**
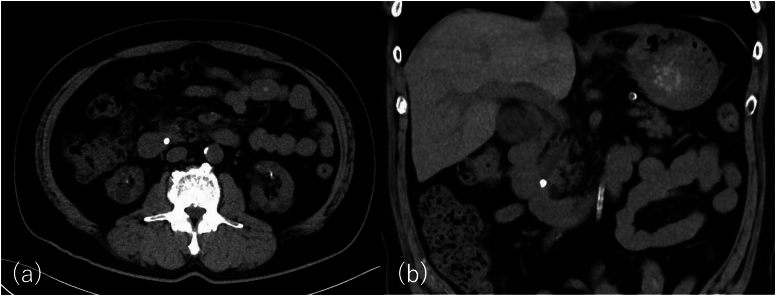
Abdominal computed tomography at the time of hospitalization. Abdominal computed tomography showing common bile duct dilation and common bile duct stone in axial (A) and coronal (B) views.

**Figure 2:**
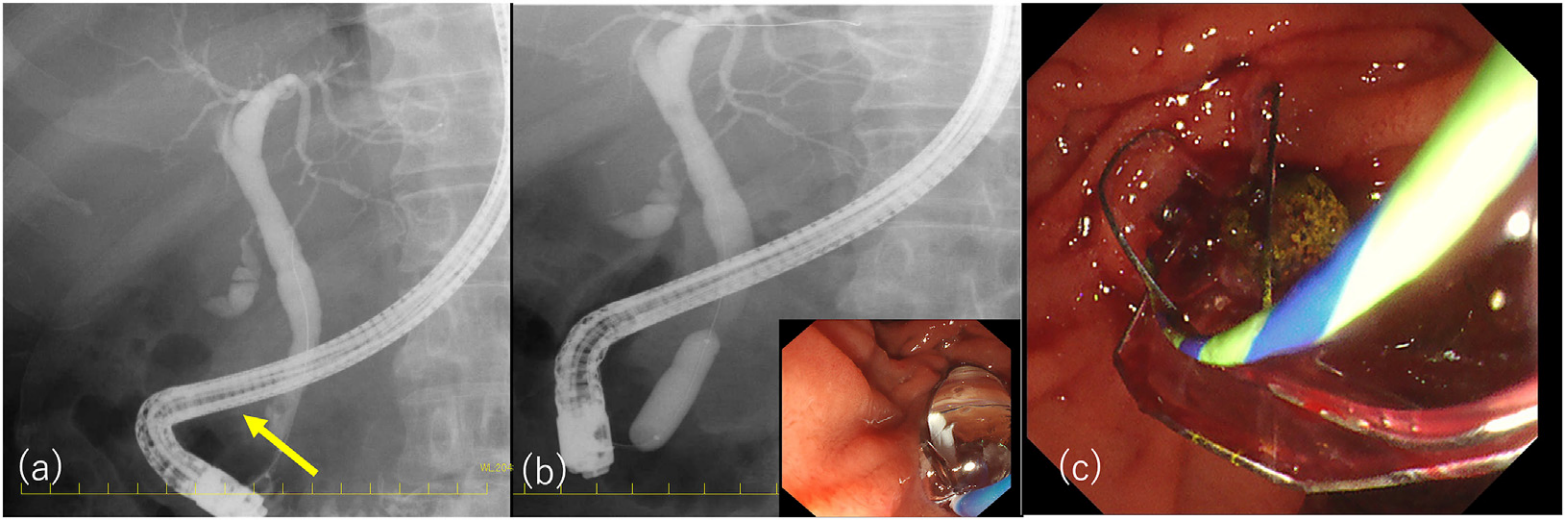
Endoscopic retrograde cholangiopancreatography images. (A) Endoscopic retrograde cholangiopancreatography showing a common bile duct stone, indicated by the yellow arrow. (B) Endoscopic papillary balloon dilation being performed with a biliary balloon dilation catheter. (C) The common bile duct stone is extracted with a basket.

**Figure 3:**
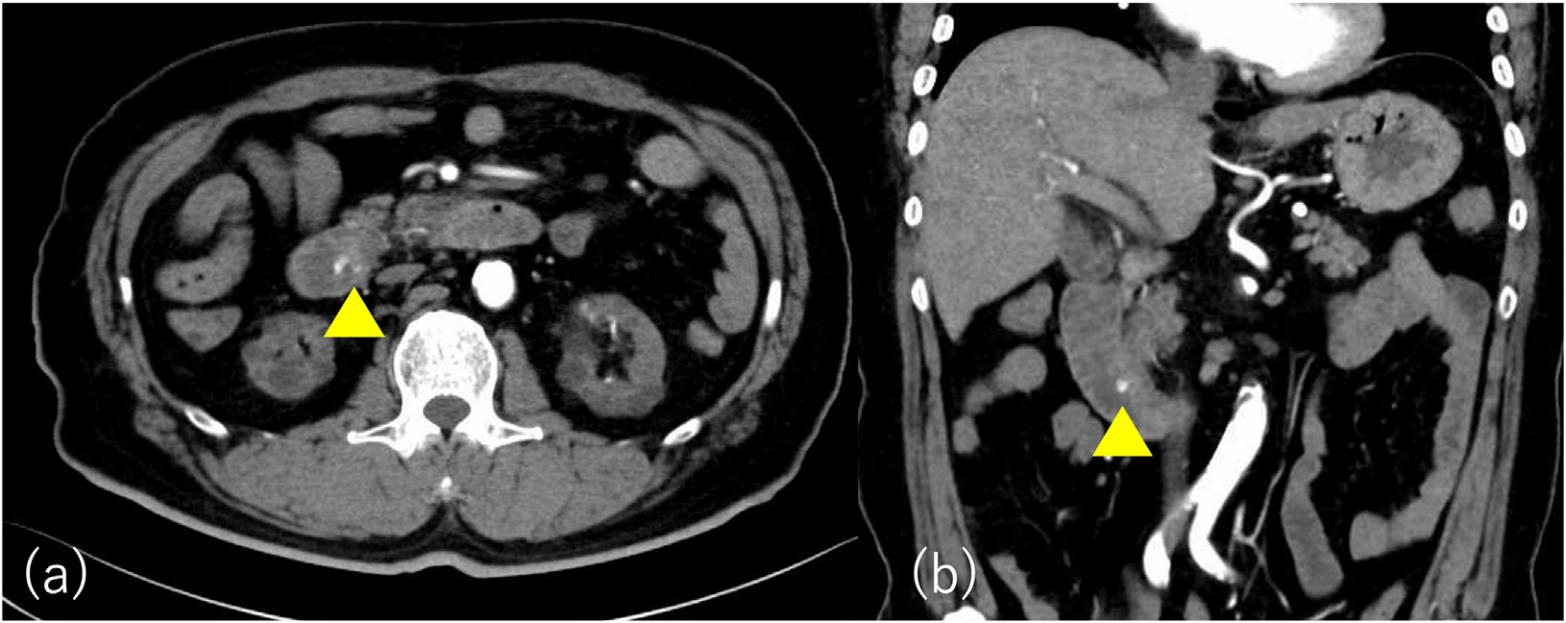
Abdominal contrast-enhanced computed tomography after hematemesis the day after the procedure. Abdominal contrast-enhanced computed tomography showing contrast leakage (yellow arrowheads) into the descending duodenal limb in axial (A) and coronal (B) views.

**Figure 4:**
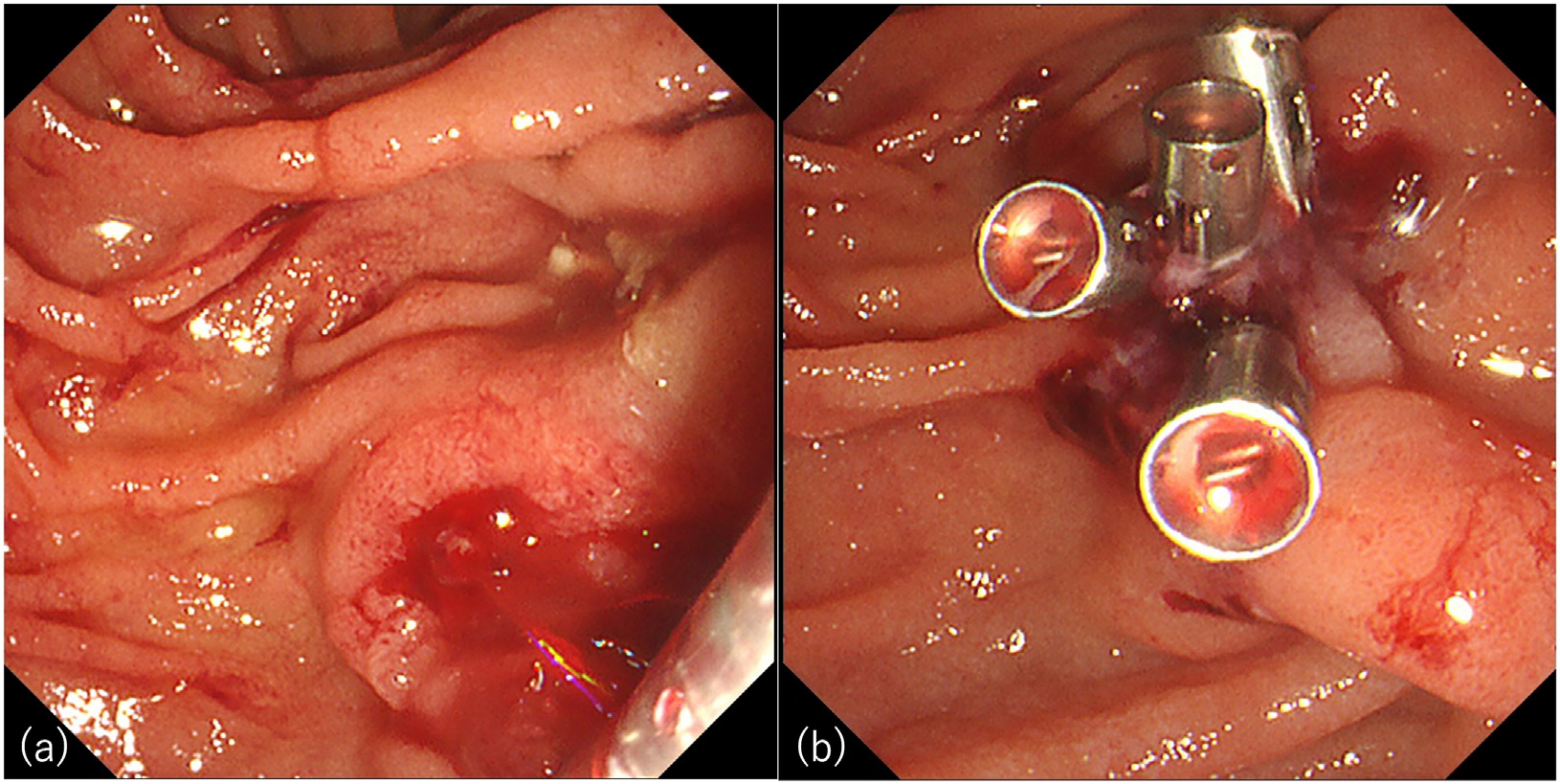
Emergency upper gastrointestinal endoscopy images. Contrast leakage into the descending duodenal limb. (A) Emergency upper gastrointestinal endoscopy showing spurting bleeding from the papillary region. (B) Clip hemostasis was performed on a visible bleeding vessel to prevent blockage of the pancreatic duct.

A 58-year-old male presented to our hospital with persistent abdominal pain. He was on maintenance dialysis for diabetic nephropathy, had a history of angina pectoris and cardiogenic cerebral embolism, and had previously undergone endoscopic retrograde cholangiopancreatography (ERCP) for symptomatic common bile duct stone disease and EPBD for stone removal. His medications included prasugrel and warfarin because of angina pectoris and cardiogenic cerebral embolism. Laboratory tests showed elevated hepatobiliary enzyme levels (aspartate aminotransferase, 174 U/L; alanine aminotransferase, 89 U/L; alkaline phosphatase, 144 U/L; gamma-glutamyl transpeptidase, 101 U/ L; total bilirubin, 0.4 mg/dL), normal level of pancreatic amylase of 39 U/L, a high white blood cell count of 8700 /mm^3^, a high C-reactive protein level of 2.1 mg/dL, and prothrombin time—international normalized ratio was 1.42. Abdominal computed tomography (CT) revealed common bile duct dilation and common bile duct stones (Figure 1). The patient was diagnosed with acute cholangitis. We considered stone removal with EST after a sufficient withdrawal period, but the acute cholangitis was mild and the stones were not large on CT, so we considered stone removal with EPBD sufficient. Then ERCP was performed (TGF-290V; Olympus Medical Systems, Tokyo, Japan). After cannulation of the common bile duct with a 5-Fr catheter (ERCP catheter; MTW Endoskopie, Wesel, Germany) and a guidewire (J-WIRE Prologue; J-MIT, Kyoto, Japan), ERCP revealed a stone, approximately 10 mm in size, in the common bile duct (Figure 2A). Although the patient was taking warfarin, his prothrombin time–international normalized ratio was within the therapeutic range. Due to the lack of a sufficient prasugrel withdrawal period, EPBD was performed instead of EST with a biliary balloon dilation catheter for 2 minutes after confirming the disappearance of the notch (REN 10–12 mm, Kaneka Medical Products, Tokyo, Japan) (Figure 2B).^6^ Subsequently, we successfully extracted the common bile duct stone using a basket (FlowerBasket V; Olympus Medical Systems, Tokyo, Japan) (Figure 2C). Blood tests the day after treatment showed no elevation of amylase and no abdominal symptoms, so the patient was not considered to have post-ERCP pancreatitis. Although abdominal pain improved, hematemesis occurred one day postprocedure. Blood tests showed that hemoglobin had dropped from 9.7g/dL to 8.0g/dL. Two units of concentrated red blood cells were transfused. Abdominal contrast-enhanced CT showed active contrast extravasation into the descending part of the duodenum, and duodenal papillary bleeding after EPBD was suspected (Figure 3). Emergency upper gastrointestinal endoscopy with a side-viewing duodenoscope (TGF-290V; Olympus Medical Systems, Tokyo, Japan) revealed spurting bleeding from the papillary region (Figure 4A). Clip hemostasis was performed on the visible bleeding vessel to prevent blockage of the pancreatic duct opening (Figure 4B). After hemostasis, the patient developed mild abdominal pain. Blood tests showed elevated pancreatic amylase levels of 995 U/L, leading to a diagnosis of mild pancreatitis. There was no pancreatic amylase elevation after stone removal, but pancreatic enzyme was elevated after hemostasis, suggesting that blocking pancreatic mouth during hemostasis caused pancreatitis. The patient's condition improved after supplemental fluids and analgesia. The patient started oral intake on the day after pancreatitis onset, experienced no further bleeding after meals began, and was discharged.

## Discussion

EST and EPBD are the standard endoscopic procedures for the treatment of bile duct stones.^1^, ^2^, ^3^, ^4^, ^5^ Although there are reports of lower incidence of pancreatitis and better stone removal rates in patients treated with EST compared to EPBD, previous reports have also shown no significant difference in the success rate of stone removal or the incidence of pancreatitis between EST and EPBD.^6^, ^7^, ^8^, ^9^ The rates of clinically significant bleeding associated with EST and EPBD have been reported to be 1%–5% and <1%, respectively.^4^^,^^10^, ^11^, ^12^, ^13^, ^14^, ^15^, ^16^ There have been no previous reports of spurting bleeding caused by EPBD. In addition, considering the non-incisional method, postprocedural bleeding is thought to be less likely with EPBD, hence its typical use in patients with bleeding tendencies. Reported risk factors for procedure-related bleeding include taking antiplatelet agents or anticoagulants, coagulopathy, chronic renal failure, and liver cirrhosis.^16^ In this case, postprocedural bleeding may have been related to antithrombotic and antiplatelet medications, maintenance dialysis due to diabetic nephropathy.

Typically, EPBD is performed using a 6–8 mm diameter dilator balloon while an endoscopic papillary large balloon dilation uses a dilator balloon with diameter ≥12 mm.^17^, ^18^, ^19^ In this case, the stone was approximately 10 mm in diameter, and we used a 10–12 mm diameter balloon, which does not meet the definition of large balloon dilatation but is somewhat larger than EPBD for stone removal. Although there is a report of dilation for 5 minutes, most reports of dilation times were less than 1 minute.^8^^,^^19^^,^^20^ In this case, the dilatation time was 2 minutes, which was slightly longer than that in the other cases. However, previous reports have found no differences in bleeding rate attributable to balloon size or dilation time.^8^^,^^19^^,^^20^

The patient developed postoperative pancreatitis. The patient had no abdominal symptoms after the first ERCP; therefore, it was thought that the pancreatitis was caused by the effect of the clip on the pancreatic duct opening during hemostasis.

EPBD, which is associated with a low bleeding risk, can cause spurting bleeding. It should be performed with caution, and the possibility of postprocedural bleeding should always be considered.
